# A Clinical Practice Guideline for Prevention, Diagnosis and Management of Intraoperative Spinal Cord Injury: Recommendations for Use of Intraoperative Neuromonitoring and for the Use of Preoperative and Intraoperative Protocols for Patients Undergoing Spine Surgery

**DOI:** 10.1177/21925682231202343

**Published:** 2024-03-25

**Authors:** Michael G. Fehlings, Mohammed Ali Alvi, Nathan Evaniew, Lindsay A. Tetreault, Allan R. Martin, Stephen L. McKenna, Vafa Rahimi-Movaghar, Yoon Ha, Steven Kirshblum, Nader Hejrati, Nisaharan Srikandarajah, Ayesha Quddusi, Ali Moghaddamjou, Anahita Malvea, Ricardo Rodrigues Pinto, Rex A. W. Marco, Virginia F. J. Newcombe, Saumayajit Basu, Samuel Strantzas, Carl M. Zipser, Sam Douglas, Ilya Laufer, Dean Chou, Rajiv Saigal, Paul M. Arnold, Gregory W. J. Hawryluk, Andrea C. Skelly, Brian K. Kwon

**Affiliations:** 1 Division of Neurosurgery and Spine Program, Department of Surgery, 7938 University of Toronto, Toronto, ON, Canada; 2 Division of Neurosurgery, Krembil Neuroscience Centre, Toronto Western Hospital, 7989 University Health Network, Toronto, ON, Canada; 3 Institute of Medical Science, 7938 University of Toronto, Toronto, ON, Canada; 4 Department of Surgery, Orthopaedic Surgery, Cumming School of Medicine, McCaig Institute for Bone and Joint Health, 2129 University of Calgary, Calgary, AB, Canada; 5 Department of Neurology, 12297 NYU Langone Medical Center, New York, NY, USA; 6 Department of Neurological Surgery, University of California, Davis, Davis, CA, USA; 7 Department of Neurosurgery, Stanford University, Stanford, CA, USA; 8 Sina Trauma and Surgery Research Center, 48439 Tehran University of Medical Sciences, Tehran, Iran; 9 Department of Neurosurgery, College of Medicine, 26721 Yonsei University, Seoul, Korea; 10 Kessler Institute for Rehabilitation, 12286 Rutgers New Jersey Medical School, Newark, NJ, USA; 11 Spinal Unit (UVM), Centro Hospitalar Universitário de Santo António, Hospital CUF Trindade, Porto, Portugal; 12 Department of Orthopedic Surgery, Houston Methodist Hospital, Houston, TX, USA; 13 Department of Medicine, University Division of Anaesthesia and PACE, 2152 University of Cambridge, Cambridge, UK; 14 Kothari Medical Centre, Kolkata, India; 15 Division of Neurosurgery, Department of Surgery, 7979 The Hospital for Sick Children, 7938 University of Toronto, Toronto, ON, Canada; 16 Spinal Cord Injury Center, 31031 Balgrist University Hospital, Zurich, Switzerland; 17 Praxis Spinal Cord Institute, Vancouver, BC, Canada; 18 Department of Neurosurgery, NYU Grossman School of Medicine, New York, NY, USA; 19 Department of Neurosurgery, Columbia University, New York, NY, USA; 20 Department of Neurological Surgery, 7284 University of Washington, Seattle, WA, USA; 21 Department of Neurosurgery, University of Illinois Champaign-Urbana, Urbana, IL, USA; 22 Department of Neurosurgery, 1077 Cleveland Clinic Akron GeneralHospital, Akron, OH, USA; 23 Aggregate Analytics, Inc., Fircrest, WA, USA; 24 Department of Orthopaedics, 8166 University of British Columbia, Vancouver, BC, Canada; 25 International Collaboration on Repair Discoveries (ICORD), 8166 University of British Columbia, Vancouver, BC, Canada

**Keywords:** intraoperative neuromonitoring, somatosensory evoked potential, motor evoked potential, electromyography, D-Wave, multimodal, intraoperative spinal cord injury

## Abstract

**Study Design:**

Development of a clinical practice guideline following the Grading of Recommendations, Assessment, Development and Evaluation (GRADE) process.

**Objective:**

The objectives of this study were to develop guidelines that outline the utility of intraoperative neuromonitoring (IONM) to detect intraoperative spinal cord injury (ISCI) among patients undergoing spine surgery, to define a subset of patients undergoing spine surgery at higher risk for ISCI and to develop protocols to prevent, diagnose, and manage ISCI.

**Methods:**

All systematic reviews were performed according to PRISMA standards and registered on PROSPERO. A multidisciplinary, international Guidelines Development Group (GDG) reviewed and discussed the evidence using GRADE protocols. Consensus was defined by 80% agreement among GDG members. A systematic review and diagnostic test accuracy (DTA) meta-analysis was performed to synthesize pooled evidence on the diagnostic accuracy of IONM to detect ISCI among patients undergoing spinal surgery. The IONM modalities evaluated included somatosensory evoked potentials (SSEPs), motor evoked potentials (MEPs), electromyography (EMG), and multimodal neuromonitoring. Utilizing this knowledge and their clinical experience, the multidisciplinary GDG created recommendations for the use of IONM to identify ISCI in patients undergoing spine surgery. The evidence related to existing care pathways to manage ISCI was summarized and based on this a novel AO Spine-PRAXIS care pathway was created.

**Results:**

Our recommendations are as follows: (1) We recommend that intraoperative neurophysiological monitoring be employed for high risk patients undergoing spine surgery, and (2) We suggest that patients at “high risk” for ISCI during spine surgery be proactively identified, that after identification of such patients, multi-disciplinary team discussions be undertaken to manage patients, and that an intraoperative protocol including the use of IONM be implemented. A care pathway for the prevention, diagnosis, and management of ISCI has been developed by the GDG.

**Conclusion:**

We anticipate that these guidelines will promote the use of IONM to detect and manage ISCI, and promote the use of preoperative and intraoperative checklists by surgeons and other team members for high risk patients undergoing spine surgery. We welcome teams to implement and evaluate the care pathway created by our GDG.

## Summary of Recommendations

### Recommendation 1

We recommend that intraoperative neurophysiologic monitoring be employed for high risk patients undergoing spine surgery.

*Quality of Evidence: Low*

*Strength of Recommendation: Strong*

### Recommendation 2

We suggest that patients at “high risk” for ISCI during spine surgery be proactively identified; that after identification of such patients, multi-disciplinary team discussions be undertaken to manage patients; and that an intraoperative protocol including the use of IONM be implemented.

*Quality of Evidence: Very Low*

*Strength of Recommendation: Weak*

## Introduction

Intraoperative spinal cord injury (ISCI) is one of the most feared complications of spine surgery and can lead to significant postoperative motor and sensory impairment.^1^ In an effort to prevent such complications, intraoperative neurophysiological monitoring (IONM) has been increasingly employed in recent years. IONM enables real-time feedback from specific nerve roots, motor tracts, and sensory tracts to measure spinal cord function intraoperatively. Currently, somatosensory evoked potentials (SSEPs), motor-evoked potentials (MEPs), and spontaneous and prompted electromyography (EMG) are the most frequently used IONM modalities for spinal procedures, either independently or in combination (multimodal neuromonitoring).^2^ Despite improvements in our understanding of IONM and its application to contemporary spine surgery, there remain considerable disagreements over the efficacy and value of using IONM in routine spine surgery cases.^3-10^ There have been previous systematic reviews with and without meta-analyses in the past, which have attempted to summarize the role of neurophysiologic monitoring for ISCI.^3-14^ However, these have focused on a specific question (for example “Diagnostic Accuracy of SSEP Changes During Lumbar Spine Surgery for Predicting Postoperative Neurological Deficit” by Chang et al^5^) or have only included comparisons of one modality vs another (for example “Diagnostic Accuracy of Combined Multimodality SSEP and Transcranial Motor Evoked Potential Intraoperative Monitoring in Patients With Idiopathic Scoliosis” by Thirumala et al^8^). A comprehensive assessment of diagnostic test accuracy (DTA) of neuromonitoring following the PRISMA-DTA guidelines and GRADE guidelines has not been performed prior to this effort. These guidelines present a high level of rigor, which enhances the clinical applicability and validity. PRISMA 2020 implementation presents several additional advantages. Readers can evaluate the applicability of the methodologies and, consequently, the veracity of the conclusions, thanks to comprehensive reporting. Healthcare professionals and policy makers can assess the relevance of the findings to their environment by presenting and summarizing the characteristics of the research that contributed to the synthesis. Policy makers, managers, and other decision makers should be assisted in developing suitable recommendations for practice or policy by describing the degree of certainty in the body of evidence supporting an outcome and the consequences of findings. Complete reporting of all PRISMA 2020 elements also makes replication and review updates easier, as well as enables teams to utilize previously completed work by including systematic reviews in overviews (of systematic reviews) and guidelines.

We developed guidelines using the GRADE approach to provide the highest evidence-based recommendations for the use of IONM for, in particular those deemed to be at higher risk for IOSCI. patients undergoing spine surgery. Based on a synthesis of the literature and a Delphi-based consensus^15^ process among members of the Guidelines Development Group (as outlined elsewhere in this Focus issue) patients at “higher risk” for ISCI were defined as those undergoing surgery for (1) complex spine deformity including a rigid thoracic curve with high deformity angular ratio (dAR); (2) revision congenital spine deformity; (3) spine conditions associated with significant cord compression and myelopathy; (4) intramedullary spinal cord tumor; (5) unstable spine fractures including those with bilateral facet dislocation and disc herniation or extension distraction injury with ankylosing spondylitis; and (6) ossification of the posterior longitudinal ligament (OPLL) associated with severe cord compression and moderate to severe myelopathy.

The systematic reviews and meta-analyses were undertaken using PRISMA standards^16^ and were registered on PROSPERO. This knowledge synthesis was conducted to summarize the evidence for the efficacy of SSEP, MEP, EMG and multimodal monitoring in detecting ISCI. Throughout this process, we sought to distinguish the specific diagnostic efficacy of neuromonitoring within subgroups of pathology, including deformity, tumor, and degenerative diseases. In addition, the existing care pathways and approaches to managed ISCI were reviewed and summarized. Based on this, a novel AO Spine-PRAXIS care pathway for the prevention, diagnosis and management of ISCI was formulated. The overarching goal of these guidelines is to standardize the use of neuromonitoring and to encourage surgeons and care teams to employ this technology in an evidence-based manner in the care of their patients.

## Methods

Clinicians from a variety of surgical and nonsurgical specialties comprised the multidisciplinary guideline development group (GDG). A rigorous conflict of interest process was undertaken for all members of the GDG, who at the outset were required to reveal any financial and intellectual interests, and to commit to the consensus-based process of GRADE. All potential conflicts were vetted in advance and discussed openly with the GDG. The GDG undertook the development of the guidelines with editorial freedom and without any influence from funding sources. To define the purpose and scope of the guideline and to steer its development, a methodology for guidelines was developed using the Conference on Guideline Standardization (COGS) checklist.^17,18^ On the basis of acknowledged methodological guidelines, systematic evaluations were carried out to compile the data supporting the suggestions. The individual evaluations in this focus issue include details about the precise techniques applied to each topic. The grading recommendations, assessments, development, and evaluation (GRADE) Working Group’s methods were used to gauge the overall quality (strength) of the evidence supporting important outcomes.^19,20^ The GRADE Guideline Development Tool was used to record the procedure, evaluate the advantages and disadvantages of different choices, and assess the strength of the recommendation.^21-24^ To generate the final recommendations for each of the issues covered, consensus sessions employing a modified Delphi methodology^15^ were held with the interdisciplinary, multinational GDGs using online video conferencing technology and anonymous voting. Consensus was defined as 80% agreement. Methodologists from Aggregate Analytics provided methodological expertise on the guideline formulation process and worked closely with clinical authors to conduct the systematic reviews. They had no financial or intellectual conflicts of interest.

### Clinical Recommendations

**Part 1**:

*Key Question:* Should we recommend intraoperative neurophysiologic monitoring for patients undergoing spine surgery deemed to be “high risk”?

 *Recommendation:* We recommend that intraoperative neurophysiologic monitoring be employed for high-risk patients

*Quality of Evidence: Low*

*Strength of Recommendation: Strong*

### Evidence Summary

A comprehensive systematic review and DTA meta-analysis was performed to assess the efficacy of neuromonitoring for detecting ISCI, following the PRISMA-DTA guidelines and GRADE guidelines. This review may be found in another article in this issue and the results are summarized below.

A total of 164 studies consisting of 99,937 patients were included. Of the 164 studies included, 16 (9.75%) were prospective while 148 (90.25%) were retrospective. In terms of disease group in the included studies, most studies included patients with mixed pathology (29.87%, n = 49), followed by deformity (26.83%, n = 44), degenerative disc disease (21.95%, n = 36), tumors (17.68%, n = 29), trauma (1.83%, n = 3), congenital diseases (1.2%, n = 2) and arteriovenous malformation (AVM) (.6%, n = 1). Most studies featured centers/hospitals from United States (35.36%, n = 58), followed by Japan (15.85%, n = 26), China (9.1%, n = 15), Korea, UK (5.5% each, n = 9), Canada, Switzerland (4.9% each, n = 8), followed by others. Most studies consisted of adult patients (50%, n = 82), followed by studies which had both adolescent and adult patients (34.7%, n = 57) and adolescents (9.1%, n = 15). Ten studies (6%) did not specify patient age. Of the 164 studies, 52 studies (31.7%) presented data for SSEPs, 75 studies (45.7%) presented data for MEPs, 16 studies (9.75%) presented data for EMG, and 69 studies (42.07%) presented data for multimodal neuromonitoring.

A total of 52 studies presented data for SSEPs, consisting of a total of 18 076 patients. Overall, the sensitivity of SSEPs was found to be 67.5% (95% CI 50.9–80.6, Heterogeneity: I^2^ = 62%, τ^2^ = 5.9269, *P* < .01), while the specificity was found to be 96.8% (95% CI 94.8–98.1, Heterogeneity: I^2^ = 95%, τ^2^ = 3.8246, *P* < .01). The I^2^ heterogeneity represents the percentage of the total variability in a set of effect sizes due to true heterogeneity, that is, to between-studies variability. Overall, the Receiver Operating Characteristics Area Under the Curve (AUC) value was found to be .899, while the Diagnostic Odds Ratio (DOR) was found to be 41.9 (95% CI 24.1–73.1).

A total of 75 studies presented data for MEPs, consisting of a total of 79 545 patients. Overall, the sensitivity of MEP was found to be 90% (95% CI 86.1-92.9, Heterogeneity: I^2^ = 32%, τ^2^ = 1.91, *P* < .01), while the specificity was found to be 95.6% (95% CI 94–96.7, Heterogeneity: I^2^ = 97%, τ^2^ = 2.7, *P* < .01). Overall, the AUC value was found to be .927, while the DOR was found to be 103.25 (95% CI 69.98–152.34).

A total of 16 studies presented data for EMG, consisting of 7004 patients. Overall, the pooled sensitivity for EMG was found to be 48.3% (95% CI 31.4–65.6, Heterogeneity I^2^ = 54, τ^2^ = 1.27, *P* < .01), while the pooled specificity was found to be 92.9% (CI 84.4–96.9, Heterogeneity I^2^ = 97, τ^2^ = 3.1, *P* < .01). The AUC was found to be .773 and the DOR was found to be 11.2 (95% CI 4.84–25.97).

A total of 69 studies with 58 325 patients presented data for any combination of multimodal neuromonitoring as outlined in detail in the systematic review on this topic in this Focus issue. Overall, the sensitivity of multimodal neuromonitoring was found to be 91% (95% 86–94.3, Heterogeneity: I^2^ = 40%, τ^2^ = 2.4511, *P* < .01), while the pooled specificity was found to be 93.8% (95% 90.6–95.9, Heterogeneity: I^2^ = 96%, τ^2^ = 3.9819, *P* > .99). The AUC value was found to be .903 while the DOR was found to be 71.97 (95% 42.17–122.8).

We also assessed publication bias for each of the groups. DTA meta-analyses differ from conventional intervention meta-analysis in several ways, making it more difficult to estimate the likelihood of publication bias. The Egger’s test is a statistical method in typical meta-analysis for identifying funnel plot asymmetry, i.e. it determines whether there is a stronger correlation between anticipated intervention effects and a study size than what would be expected to happen by chance.^25^ In order to test the global null hypothesis that “all of the univariate funnel plots for multiple outcomes are symmetric,” Hong et al (2020) first proposed an expanded version of this test for multivariate meta-analysis.^26^ In comparison to the common univariate publication bias test, this overall test contains various outcome information, and the statistical power is often increased. The Hong’s test (also known as MSSET) avoids correlation data among various outcomes that is occasionally absent under certain circumstances of multivariate meta-analysis. However, for DTA meta-analysis, the Reitsma’s bivariate meta-analysis model has all of the correlation data, and since MSSET does not make use of this data, its statistical power may be wasteful.^27^ For the same global null hypothesis, Noma (2020) created an alternative generalized Egger’s tests that successfully take into account the correlation data (called as MSSET2 and MSSET3). Because Noma’s tests make use of correlation data, it is anticipated that they will have greater statistical power than the MSSET when applied to DTA meta-analysis.

For SSEP neuromonitoring, we observed slight asymmetry and the weighted regression with multiplicative dispersion test for asymmetry was not found to be statistically significant (t = 1.61, df = 60, *P* = .11). For MEP neuromonitoring, we observed asymmetry and the weighted regression with multiplicative dispersion test for asymmetry was found to be statistically significant (t = 4.42, df = 92, *P* < .001). For multimodal neuromonitoring, we observed asymmetry and the weighted regression with multiplicative dispersion test for asymmetry was not found to be statistically significant (t = .72, df = 15, *P* = .48). For multimodal neuromonitoring**,** we observed asymmetry and the weighted regression with multiplicative dispersion test for asymmetry was also found to be statistically significant (t = 5.03, df = 79, *P* < .001).

For SSEP monitoring, of the 52 studies, 10 studies (19.2%) were found to have “some concerns” as per the risk of bias assessment part of the QUADAS tool, 25% (n = 13) were found to be “high risk” and the remaining 29 studies (55.8%) were found to be “low risk.” For most of the studies that were graded down, the particular domain was “reference standard”; the reason was either lack of specification/details of the postoperative examination used, or use of a non-standard exam. For MEP monitoring, of the 75 studies, 21 studies (28%) were found to have some concerns, 10.7% (n = 8) were found to be high risk and the remaining 46 studies (61.3%) were found to be low risk. For most of the studies that were graded down, the particular domain was “reference standard.” For EMG monitoring, of the 16 studies, three studies (18.75%) were found to have some concerns, 25% (n = 4) were found to be high risk and the remaining nine studies (56.25%) were found to be low risk. For most of the studies that were graded down, the particular domain was “index test”; the reason was lack of specification/details of the changes in EMG monitoring that were considered an alert.

For multimodal neuromonitoring, of the 69 studies, 14 studies (20.3%) were found to have some concerns, 14 studies (20.3%) were found to be high risk and the remaining 41 studies (59.4%) were found to be “low risk.” For most of the studies that were graded down, the particular domain was “index”; the reason was lack of specification/details of the criteria that constituted an alert.

We applied the GRADE assessment methodology pertinent to DTA meta-analysis to evaluate the strength of evidence for each of the four groups, i.e. SSEP, MEP, EMG and multimodal neuromonitoring. For all four groups, the final quality of the evidence was found to be “Low.” Evidence was downgraded particularly for “Inconsistency,” “Imprecision” and “Publication Bias.” The inconsistency score was downgraded because of differences in included population/pathology type (deformity vs tumor vs degenerative vs mixed population) and because of use of different “thresholds.” “Imprecision” was downgraded due to a low number of events (true positives + false negatives) resulting in large confidence intervals, particularly for sensitivity. Finally, “Publication Bias” was downgraded due to both observed and statistically significant asymmetry.

### Rationale for Recommendation

During the consensus meeting held via virtual video-teleconferencing, the GDG reviewed the evidence and results of the meta-analysis, and then went through the Evidence-to-Decision framework with anonymous voting to address each of the considerations necessary for making the recommendation. Consensus was defined as 80% agreement. The GDG agreed (92% Yes and 8% probably yes) that ISCI is indeed a high priority problem, given that the incidence of new deficit may be up to 23% for deformity surgery and 61% for tumor surgery.^27^ Moreover, ISCI may be associated with significant morbidity for the patient and their caregivers, and with significant liability burden for the surgeon and care team.

The GDG agreed (100% consensus) that the desirable anticipated effects are large, given that implementing neuromonitoring has been shown to reduce the risk of injury; that even if injury does occur, the potential opportunity to reverse or minimize the underlying neurologic deficit is higher, and that having neuromonitoring alerts can prompt care teams to put treatment algorithms into motion.

The GDG agreed that the undesirable effects of neuromonitoring are small (100% consensus). These effects include the need for neuromonitoring equipment, availability of neurophysiologist/technologists for procedures, time to set up the equipment intraoperatively, and a certain degree of unnecessary disruption due to false alerts.

The GDG agreed that the certainty of evidence of the systematic review and meta-analysis is moderate (83% moderate, 8.5% low, 8.5% high). Based on our DTA meta-analysis, most included studies in the analyses were low risk as assessed using QUADAS. However, when applying the GRADE assessment scoring, strength of evidence was downgraded particularly for “Inconsistency,” “Imprecision” and “Publication Bias.” The inconsistency score was downgraded because of differences in included population/pathology type (deformity vs tumor vs degenerative vs mixed population) and because of the use of different “thresholds.” “Imprecision” was downgraded due to low number of events (true positives + false negatives) resulting in large confidence intervals, particularly for sensitivity. Finally, “Publication Bias” was downgraded due to both observed and statistically significant asymmetry.

The GDG agreed that there is either no (64%) or possibly no (27%) important uncertainty or variability in how much all stakeholders value the main outcome, given that reduction of neurologic injury during spine surgery is important to all stakeholders.

The GDG agreed (82%) that the balance between desirable and undesirable effects probably favors the intervention, given that the risk of injury with no monitoring overweighs the resource/technical challenges associated with neuromonitoring.

Most of the GDG members agreed that resource requirements, i.e. costs associated with neuromonitoring are moderate (90% moderate, 10% negligible costs or savings). These include the cost of the required equipment as well as that of the neurophysiologist/technician and increased OR times. The evidence related to the source requirement and costs, unfortunately, does not exist. The GDG acknowledged this and identified this as a knowledge gap that future studies should investigate.

Most of the GDG members agreed that the cost effectiveness probably favors the intervention (82% probably favors the intervention, 9% probably favors the comparison and 9% favors the intervention). According to a study by Sala et al^28^, IONM may be cost-effective provided the expenditures do not exceed $977 per surgery, based on a reported paraplegia rate of .1% in young people after scoliosis surgery and taking lifetime healthcare costs into consideration. However, the authors’ analysis model assumed that IONM completely prevents all injuries (100 percent prevention rate). The potential indirect costs of erroneous IONM notifications were not taken into account. As many spine surgeons have experienced the heightened anxiety caused by IONM notifications, it was acknowledged that erroneous “false positives” certainly can have a negative impact on the case, and not being able to factor that in quantitatively is a limitation of the current literature.

The GDG agreed that if IONM were to be utilized broadly that health inequity will be reduced (100% consensus), as it is currently only offered in well-resourced regions and high-income countries. Guidelines and policy change will likely help extend these technologies to low-income countries.

The GDG also agreed that a recommendation for monitoring for high-risk patients will probably be acceptable (100% consensus) to clinicians under the important caveat that appropriate resources are available. The GDG also agreed that a recommendation for monitoring will reduce the risk of ISCI with some additional cost but significant opportunity for long-term saving/reduced liability. The GDG agreed that the feasibility of implementing this intervention may vary (82% varies, probably varies 18%) given the challenges in implementing this in low-income countries and that remote centers may not have access to personnel or the equipment.

Despite the low quality of the evidence, the strength of recommendation was strong. This is because GRADE has separate frameworks to judge the quality of evidence and strength of recommendation. In fact, the “quality” of the evidence is just one component that determines the “strength” of recommendation. Other factors, as highlighted above include considerations for perceived benefits/harms, the values, feasibility, implications of equity, associated with making/not making the recommendation. Hence, if all other factors are accepted by the GDG to strongly weigh in favor of making the recommendation, this may mitigate the impact of low quality of the evidence on the strength of recommendation.

#### Recommendation

Based on these explanations, most GDG members (82%) agreed that the desirable consequences *clearly* outweigh undesirable consequences in most settings and *recommended* that neuromonitoring should be offered (91%) for “high-risk” patients.

**Part 2**:

Ke*y Question:* Should we recommend that patients at “high risk” for ISCI during spine surgery be proactively identified, that after identification of such patients, multi-disciplinary team discussions be undertaken to manage patients, and that an intraoperative protocol including the use of IONM be implemented?

*Recommendation:* We suggest that patients at “high risk” for ISCI during spine surgery be proactively identified, that after identification of such patients, multi-disciplinary team discussions be undertaken to manage patients, and that an intraoperative protocol including the use of IONM be implemented.

*Quality of Evidence: Very Low*

*Strength of Recommendation: Weak*

### Evidence Summary

Evidence considered for this recommendation was derived from the scoping review on the Definition, Frequency and Risk Factors for ISCI and the scoping review on the Management of ISCI, which are included in preceding manuscripts within this Focus Issue. Six studies evaluated the risk of an ISCI, four of which reported risk factors for neurological deficits in the immediate postoperative period using changes in ASIA grades (Fehlings 2018,^29-31^ Chen 2012,^29-31^ Romero-Munoz 2019,^29-31^ Zhang 2017^32^) and one study using a definition of “any new limb, motor, or sensory deficit” (Kim 2021^33^). One study evaluated the risk for ISCI using a ≥50% drop in SSEP and/or MEP amplitudes (Buckland 2018^34^). Risk of bias of nonrandomized studies was assessed using the Quality in Prognosis Studies (QUIPS) tool for studies evaluating risk factors.^35^ Based on the risk of bias assessment, studies were rated as “good,” “fair” or “poor” quality. Evidence for risk factors for neurological deficits in patients with deformities originate from one good-quality, prospective cohort (N = 265) (Fehlings 2018)^29-31^ studying an adult scoliosis patient population, and one fair-quality, retrospective cohort (N = 62) in patients with congenital scoliosis (19%), kyphoscoliosis (74%), and kyphosis (7%). Another poor-quality retrospective cohort (N = 2210) (Buckland 2018)^34^ described risk factors for intra-operative neuromonitoring alerts in adolescent patients with idiopathic scoliosis. For patients with “mixed” indications, one good-quality, retrospective cohort (N = 316) (Chen 2012)^29-31^ reported on patients with spinal degeneration (35%), tumor (23%), trauma (22%), deformity (16%), and inflammation (4%), while one fair-quality retrospective cohort (N = 1282) (Romero-Munoz 2019)^29-31^ reported on patients presenting with spinal degeneration (75%), deformity (18%), fractures (4%), and other rare injuries (4%). Common methodological concerns included retrospective collection of complications (five of the six studies were retrospective study designs) and unclear or unknown study attrition. Other less frequent concerns included inadequate description of inclusion/exclusion criteria, unclear validity and/or reliability of the measurement methods for prognostic factors and/or confounders.

A total of 21 risk factors were explored which were broadly categorized into patient–related (eg demographics and comorbidities), clinical (eg preoperative neurological status and presence of myelopathy), surgical (eg number of surgical levels, the use of osteotomies) and radiological risk factors (eg coronal dAR, curve magnitude).

With regard to patient demographics, older age was found to have increased odds for ISCI in two studies (Zhang 2017,^32^ Fehlings 2018^29-31^) in patient cohorts with spinal deformities (OR = 1.53 [95% CI 1.13–2.06], *P* = .0.05; and OR = 8.27 [95% CI 1.17–58.71], *P* = .035) and in one study (Chen 2012)^29-31^ with a mixed patient population consisting of patients with spinal degeneration, tumors, trauma, deformity, and inflammation (OR = 1.08 [95% CI 1.03–1.13], *P* < .001). Due to inclusion of different age groups and varied methods of age modeling (i.e. continuous vs categorical), the magnitude of effect varied across studies reporting an association. While gender was associated with increased odds of ISCI in one study (Chen 2012)^30^ in a mixed patient population with different underlying spinal pathologies (OR 5.22 [95% CI 1.86–14.62), *P* = .0002), no association was seen in a smaller study (Kim 2021)^33^ in patients with degenerative disease (OR = 1.378 [95% CI 0.22–5.79], *P* = .661). Across two studies (Chen 2012,^30^ Romero-Munoz 2019^31^) in patient populations with mixed spinal pathologies, hypertension was not consistently associated with increased odds of SCI (OR = 15.18 [95% CI 4.5–51.17], *P* < .001^30^; and OR = 1.47 [95% CI 0.56–3.86], *P* = .436). Abnormal pulmonary function may increase the odds of ISCI (OR = 2.1 [95% CI 0.99–4.48], *P* = .054), (Zhang 2017^32^). Other patient-related factors (including diabetes, obesity, BMI, presence of depression, Charlson-Comorbidity Index and dyslipidemia) were shown to not significantly increase a patient’s individual risk for an ISCI during spinal surgery.

From a clinical perspective, one study (N = 62, Zhang 2017)^32^ in patients with congenital scoliosis, kyphoscoliosis and kyphosis found no association between preoperative AIS and neurological deficits (OR: NR, *P* > .05), while another study (N = 316, Chen 2012)^30^ in mixed populations that included spinal degeneration, tumors, trauma, deformity, and inflammation found decreased odds for ISCI in patients with a better pre-operative AIS grade (OR = .35 [CI 0.18–.66], *P* = .001). One study (N = 196, Kim 2021)^33^ found OPLL with combined myelopathy to be associated with increased odds of neurological deficit (OR = 8.24 [CI 1.57–43.38], *P* = .013).

In terms of surgery-related factors, no statistically significant associations were reported between the number of spinal levels operated and rates of ISCIs in patients with scoliosis (Fehlings 2018, N = 265; OR = 1.08 [95% CI 0.99–1.17], *P* = .091)^29-31^ and in patients with OPLL (Kim 2021, N = 196; OR = 1.36])^33^, but an increasing number of operated segments was associated with significantly higher odds of SCI in another study that included patients with mixed pathologies (Chen 2012, N = 316; OR 3.28 [95% CI 1.55–6.92], *P* = .002).^30^ One study showed that the use of IONM during surgery for OPLL greatly decreases the risk for ISCIs (Kim 2021, N = 196; OR = .14 [95% CI).^33^ The study by Fehlings et al^29-31^ found a statistically significant increase in odds for postoperative neurological deficits in adults with scoliosis if patients received lumbar-level osteotomies (OR = 3.3, [95% 1.18–9.17], *P* = .022). These deficits included cauda equina injury, which would be considered a type of ISCI, and isolated nerve root injuries, which are a distinct entity. However, for ease of analysis, the overall rate of neurological injury was considered. Similarly, the study by Buckland et al (N = 2210)^34^ showed significantly higher rates of intraoperative neuromonitoring alerts in adolescent patients diagnosed with scoliosis who underwent a Ponte-osteotomy (OR: NR, *P* < .001). Interestingly, performing a three-column osteotomy was not associated with an increased risk for ISCI. Finally, no significant associations between ISCIs and type of operation (emergency vs elective for degenerative disease) and duration of surgery were demonstrated.

Only two studies reported on radiographic risk factors for ISCIs. Fehlings et al, (N = 265, 2018)^29-31^ found greater odds of postoperative neurological deficits per 1 unit increase of coronal DAR in scoliosis patients undergoing deformity correction (OR = 1.1 [95% CI 1.01–1.19], *P* = .037). The DAR measures the acuteness of the curve and is defined as the maximum curve Cobb angle divided by the number of involved vertebral levels.^27^ The study by Buckland et al (N = 2,210, 2018)^34^ found an association between spinal curve magnitude and IONM alerts in patients with adolescent scoliosis but did not report an effect estimate.

The GDG agreed that the following sub-entities of spinal pathologies are deemed high risk for the occurrence of an ISCI: (i) Rigid thoracic curve with high DAR; (ii) Revision surgery for congenital deformity with significant cord compression and myelopathy; (iii) extrinsic lesions with cord compression and myelopathy; (iv) intramedullary tumors; (v) unstable fractures, (eg bilateral facet dislocations and disc herniation); (vi) extension-distraction type injury in patients with ankylosing spondylitis; and (vii) OPLL with severe cord compression and moderate to severe myelopathy. It is recognized that patients with extrinsic lesions associated with cord compression and myelopathy represent a broad category that is open to interpretation. The decision as to which patients represent “high risk” in this category has been left open to clinical judgement and is an area in which further research will be required.

The overall quality of evidence for ISCI risk factors as assessed per GRADE was low or very low for most factors across surgical conditions. Increased odds for ISCI varied by underlying pathology (eg, deformity). In patients undergoing surgery for spinal deformity, there was moderate evidence of increased risk for ISCI in patients with older age and increasing coronal DAR. There was moderate evidence that estimated blood loss and the number of spinal levels involved were not associated with increased risk of ISCI in the deformity population. There was moderate evidence that better pre-operative AIS grades were associated with decreased risk of ISCI in patient cohorts with mixed pathologies.

Although there is a paucity of quantitative results, and thus evidence, on comparative effects and harms of treatment strategies following an ISCI event, management of intraoperative signal loss and possible SCI merits a standardized protocol and care pathway to avoid and minimize the risk of postoperative neurologic deficits. This has been understood as a key knowledge gap and as a result, a number of studies, including professional organizations (such as the Scoliosis Research Society) have come together to generate care pathways and treatment algorithms in response to IONM alerts. A summary of the literature pertaining to treatment protocols and care pathways for ISCIs is provided in the scoping review entitled “The Management of ISCI - A Scoping Review.” Briefly, we identified 16 studies reporting on management methods for ISCI of which 8 were retrospective cohort studies, and two were publications of consensus meetings held using the Delphi technique. The final six studies were narrative evaluations with recommendations for intraoperative checklists and IONM alert handling procedures. Notably, 56% of the studies that were included exclusively examined patients undergoing surgery for spinal deformities. Most studies emphasized anesthesiologic, neurophysiological/technical, and surgical treatment strategies as intraoperative considerations and actions taken in the event of an ISCI.

Using the information gleaned from our scoping review, we designed a novel care pathway called the “AO Spine Praxis Care Pathway to Manage Patients at High Risk for Intraoperative Spinal Cord Neurologic Deterioration” consisting of five section: (i) initial clinical assessment, (ii) preoperative planning, (iii) surgical/anaesthetic planning, (iv) intraoperative management, and (v) postoperative management. It is important to emphasize that Steps 1, 2, and 3 of the care pathway highlight preventative steps that can be implemented before the operation to lower the risk of an ISCI happening. For intraoperative management, the first suggestion is to pause, alert the team, and remove outside distractions in order to take control of the operating room and force everyone engaged to prioritize and focus on the problem. Subsequently, to reverse the signal loss, reversible surgical, neurophysiological and anesthetic factors should be investigated. The care pathway also integrates key post-operative management strategies, including a monitored step-down or ICU bed, serial neurological functional examinations, consideration of pharmacological intervention with methylprednisolone, hemodynamic management with maintenance of mean arterial blood pressure (MAP) parameters, and the use of post-operative imaging including CT and MRI, as clinically indicated.

### Rationale for Recommendation

With a reported incidence of ISCIs of up to 23% in patients undergoing deformity surgery and up to 61% in patients undergoing surgery for intramedullary spinal cord tumors, the GDG agreed that ISCI is a priority and that risk factors, planned three-column osteotomies, high coronal DAR’s and curve magnitudes need to be identified and considered in patients undergoing spinal surgery.

Similar to the previous question, the GDG agreed (93% consensus) that the desirable anticipated effects of implementing the use of IONM for high risk spine cases would be large, given that a limited number of studies have shown that implementation of IONM reduces the risk of injury; and that even if injury occurs, opportunity to reverse IONM signal loss is higher and that having IONM alerts can prompt care teams to put treatment algorithms into motion.

The GDG voted that undesirable effects of neuromonitoring were small (64%) or trivial (36%). These effects include the requirement of neuromonitoring equipment, availability of neurophysiologists/technologists for procedures, and the potential of unnecessary disruption due to false positive alerts. No studies have explored whether identification of risk factors, implementation of multidisciplinary team assessments, and implementation of an intraoperative treatment protocol reduces the risk of ISCI. As a result, the GDG decided that the overall certainty of the evidence of effects is low. Reduction of neurologic impairment is considered of high importance to all stakeholders and as such the GDG agreed that there are no important uncertainties or variabilities in how much people value the main outcome, i.e. risk reduction for postoperative neurological deficits.

There was unanimous agreement within the GDG that the balance between desirable and undesirable effects favors the intervention, since the risk of ISCI without identifying high-risk patients, not conducting multidisciplinary team discussions and employing intraoperative treatment protocols in response to an ISCI is thought to outweigh the associated potential costs, availability of resources and technical challenges.

While it is understood that the implementation of IONM and the employment of a neurophysiologist/technician is associated with costs, there has been uncertainty as to how the costs are subdivided and to what extent generation and implementation of a checklist contributes to overall resource requirements. These uncertainties are reflected in the GDG’s votes, which included 21% votes for moderate costs, 7% votes for moderate cost savings while 64% voted that resource requirements vary and cannot be generalized, and 7% did not know what resources would be required. To the GDG’s knowledge, there have been no published studies to date investigating the financial implications of using IONM and implementing intraoperative treatment algorithms. Therefore, there was high agreement (92%) that no studies were available to support an assessment of the resources required to implement IONM protocols for high-risk spine cases.

Increasing evidence and general consensus among experts underscores the importance of spinal cord monitoring and its potential to detect impending injury in time for corrective measures to be taken, thereby increasing the likelihood of preventing or limiting a neurological deficit. However, contemporary data on the benefits of monitoring are limited to Class IV and Class III evidence. Lifetime costs of postoperative neurological deficits, which, depending on the computational model (eg direct health care costs, loss of wages/benefits) and the degree of injury, can be staggering.^36^ Healthcare costs for patients with neurological deficits secondary to spinal cord lesions mainly originate from the field of traumatic SCI, with lifetime costs for high cervical quadriplegia (C1-4) incurred at the age of 25 estimated at 5.1 million USD.^37,38^ A theoretical model using a Monte Carlo simulation concluded that intraoperative monitoring would be cost-saving for spinal surgeries using a reference case of a 50-year-old with a neurologic complication rate of 5% and a 52.4% prevention rate given an IONM alert at 94.3% sensitivity and 95% specificity, assuming incomplete motor injury.^39^ However, Class I and II studies are not available to date and are likely not to occur for both medico-legal and ethical reasons. Given the paucity of evidence, the GDG voted that the cost effectiveness of the intervention probably favors (69%) and favors (31%) the intervention.

Given its associated costs and the need for infrastructure and trained personnel, the use of IONM is commonly confined to well-resourced, high-income countries. The GDG agreed (86% consensus) that the implementation of guidelines and policies may set benchmarks that have the potential to promote low- and middle income countries (LMICs) toward reaching their goals of implementing IONM and treatment protocols; this would have the end-effect of probably reducing health inequity. Two-thirds (67%) of the GDG voted that the provision of a recommendation for identifying high risk patients preoperatively, having multidisciplinary team discussions for such high-risk patients, and implementing intraoperative protocols will probably be acceptable to key stakeholders (33% voted yes), if appropriate resources are available. It was discussed that such a recommendation will be associated with additional cost but may constitute a significant opportunity for long-term saving and reduced liability. Given the potential challenges related to limited resources (eg financial, equipment, personnel) in remote areas and LMICs, 71% of the GDG voted that the feasibility of such a recommendation varies, while 7% voted uncertain, 7% probably yes, and 14% yes.

#### Recommendation

Given the available literature and based on consensus-based discussions, most GDG members (93%) agreed that desirable consequences clearly outweigh undesirable consequences in most settings and recommended that patients at “high risk” for ISCI during spine surgery be proactively identified, that after identification of such patients, multi-disciplinary team discussions be undertaken to manage patients, and that an intraoperative protocol including the use of IONM be implemented. It was recognized that key knowledge gaps exist including validation of what constitutes a “high risk spine case” and the costs/logistical issues involved in implementing IONM protocols for high-risk spine case.

## Conclusion

In the current guidelines document, we have recommended that some form of neuromonitoring be implemented for “high risk” patients undergoing spine surgery. We have suggested that patients at “high risk” for ISCI during spine surgery be proactively identified, that after identification of such patients, multi-disciplinary team discussions be undertaken to manage patients, and that an intraoperative protocol including the use of IONM be implemented. We believe that these guidelines will influence clinical practice and will also facilitate evidence-based decision making. We acknowledge that literature is limited for the use of intraoperative checklists, and we hope that these guidelines will result in the increased use of such checklists. We also acknowledge that given that literature related to cost-effectiveness of use of IONM is limited, global adaptation and implementation of these guidelines, particularly in resource-poor areas, may be a challenge.
